# Scaffold-Type Structure Dental Ceramics with Different Compositions Evaluated through Physicochemical Characteristics and Biosecurity Profiles

**DOI:** 10.3390/ma14092266

**Published:** 2021-04-27

**Authors:** Mihai M.C. Fabricky, Alin-Gabriel Gabor, Raluca Adriana Milutinovici, Claudia Geanina Watz, Ștefana Avram, George Drăghici, Ciprian V. Mihali, Elena-Alina Moacă, Cristina Adriana Dehelean, Atena Galuscan, Roxana Buzatu, Virgil-Florin Duma, Meda-Lavinia Negrutiu, Cosmin Sinescu

**Affiliations:** 1 Department of Prosthodontics, Faculty of Dental Medicine, Victor Babeș University of Medicine and Pharmacy, 9 Revolutiei 1989 Ave., 300070 Timisoara, Romania; fabricky@me.com; 2 Department of Prostheses Technology and Dental Materials, Faculty of Dental Medicine, Victor Babes University of Medicine and Pharmacy of Timisoara, 9 Revolutiei 1989 Ave., 300070 Timisoara, Romania; alin.gabor@umft.ro (A.-G.G.); negrutiu.meda@umft.ro (M.-L.N.); minosinescu@gmail.com (C.S.); 3 Research Center in Dental Medicine Using Conventional and Alternative Technologies, Timisoara, 9 Revolutiei 1989 Ave., 300070 Timisoara, Romania; cadehelean@umft.ro; 4 Department of Orthodontics, Faculty of Dental Medicine, Victor Babeș University of Medicine and Pharmacy, 9 Revolutiei 1989 Ave., 300070 Timisoara, Romania; raluca_balan22@yahoo.com; 5 Department of Pharmaceutical Physics, Faculty of Pharmacy, Victor Babeș University of Medicine and Pharmacy, 2nd Eftimie Murgu Sq., 300041 Timișoara, Romania; 6 Research Center for Pharmaco-Toxicological Evaluations, Faculty of Pharmacy, “Victor Babes” University of Medicine and Pharmacy, Eftimie Murgu Square No. 2, 300041 Timisoara, Romania; stefana.avram@umft.ro (Ș.A.); draghici.george-andrei@umft.ro (G.D.); alina.moaca@umft.ro (E.-A.M.); 7 Department of Pharmacognosy, Faculty of Pharmacy, Victor Babeș University of Medicine and Pharmacy, 2nd Eftimie Murgu Sq., 300041 Timișoara, Romania; 8 Departament of Toxicology, Faculty of Pharmacy, Victor Babeș University of Medicine and Pharmacy, 2nd Eftimie Murgu Sq., 300041 Timișoara, Romania; 9 Department of Life Sciences, Faculty of Medicine, Vasile Goldis Western University of Arad, 86 No., Liviu Rebreanu St., 310414 Arad, Romania; mihaliciprian@yahoo.com; 10 Molecular Research Department, Research and Development Station for Bovine, 32 No., Bodrogului St., 310059 Arad, Romania; 11 Department of Preventive Dentistry, Faculty of Dental Medicine, Victor Babeș University of Medicine and Pharmacy, 14A Tudor Vladimirescu Ave., 300173 Timisoara, Romania; atedent@yahoo.com; 12 Translational and Experimental Clinical Research Center in Oral Health (TEXC-OH), 14A Tudor Vladimirescu Ave., 300173 Timisoara, Romania; 13 Department of Dental Aesthetics, Faculty of Dental Medicine, Victor Babeș University of Medicine and Pharmacy, 300041 Timişoara, Romania; 14 3OM Optomechatronics Group, Faculty of Engineering, “Aurel Vlaicu” University of Arad, 77 Revolutiei Ave., 310130 Arad, Romania; dumavirgil@yahoo.co.uk; 15 Faculty of Mechanical Engineering, Polytechnic University of Timisoara, 1 Mihai Viteazu Ave., 300006 Timisoara, Romania

**Keywords:** augmentation ceramic products, ceramic scaffolds, network porosity, elemental composition, cytocompatibility, HGF, HET–CAM

## Abstract

The design and development of ceramic structures based on 3D scaffolding as dental bone substitutes has become a topic of great interest in the regenerative dentistry research area. In this regard, the present study focuses on the development of two scaffold-type structures obtained from different commercial dental ceramics by employing the foam replication method. At the same time, the study underlines the physicochemical features and the biological profiles of the newly developed scaffolds, compared to two traditional Cerabone^®^ materials used for bone augmentation, by employing both the in vitro Alamar blue proliferation test at 24, 48 and 96 h poststimulation and the in ovo chick chorioallantoic membrane (CAM) assay. The data reveal that the newly developed scaffolds express comparable results with the traditional Cerabone^®^ augmentation masses. In terms of network porosity, the scaffolds show higher pore interconnectivity compared to Cerabone^®^ granules, whereas regarding the biosafety profile, all ceramic samples manifest good biocompatibility on primary human gingival fibroblasts (HGFs); however only the Cerabone^®^ samples induced proliferation of HGF cells following exposure to concentrations of 5 and 10 µg/mL. Additionally, none of the test samples induce irritative activity on the vascular developing plexus. Thus, based on the current results, the preliminary biosecurity profile of ceramic scaffolds supports the usefulness for further testing of high relevance for their possible clinical dental applications.

## 1. Introduction

Ceramics as a major class of dental biomaterials employed for tooth reconstruction are derived from the Greek word *keramos*, which translates to *burnt stuff* [1].

Over the years, the application of ceramics in dentistry has developed widely in response to several market innovations that patients are becoming more and more interested in. However, the success of these novel dental solutions is in fact correlated with the dental needs of the increasing rise in the elderly population, especially referring to European society.

Thus, in 2013, the European market for dental bone grafting materials was worth around USD 200 million, and it is expected that by 2024 the global market for bone substitutes will exceed the value of USD 900 million [2].

It is thought that the rapid development of market value is mainly caused by two types of structures: (i) ceramic materials—considered valuable biomaterials in the field of dental bone substitutes as, unlike metals or polymers, ceramic structures do not present a limited integration with the surrounding tissue [3]. Moreover, scaffold-type structure ceramics provide a good pattern for cell migration, thus supporting the osteoconduction phenomenon [4]; (ii) bovine derivatives—frequently used as bone grafting materials [5] due to their major advantage in generating cell proliferation, a process known as osteoinduction [4].

Nevertheless, ceramic materials have acquired their merit due to their high biocompatibility and proper physicochemical features suitable for clinical use, providing good mechanical properties, optimal thermal and optical characteristics and chemical inertness [1,6].

Therefore, these structures are used in a wide range of dental applications, from crowns and veneers up to ceramic scaffolding [7,8,9].

Three-dimensional (3D) ceramic scaffolds represent a valid approach in complete or partially edentulous patients who present an insufficient alveolar bone, in which case bone addition or augmentation prior to implant placement is frequently needed [10,11].

For small bone additions, classic augmentation materials (bovine bone, hydroxyapatite, etc.) [12] are indicated for use. However, when the bone defect is large, the use of scaffold-type structures is required in order to maintain the bone supply both, vertically and horizontally [13].

3D scaffolds can be produced through different technologies, as described in the literature: the foaming method, starch consolidation, sponge replication, solid free form fabrication [3], 3D-printing and bioprinting [14] or other techniques. Regardless of the method employed, the process itself may be considered a big challenge, since a successful scaffold must meet several important features, such us: (i) good biocompatibility; (ii) predictable biodegradability in the case of resorbable structures that should correlate with the bone regeneration rate; (iii) optimal porosity to facilitate cell proliferation and migration, but also to promote the micronutrient flux at the site; (iv) proper surface topography; (v) suitable physicochemical characteristics to obtain tailored structures for the various specific needs of the affected bones [15,16,17].

Among the most widely used ceramics/bioceramics employed for dental tissue engineering, calcium phosphate-based structures are constantly being developed due to their osteoconduction and osteoinduction features [18]. However, glass-ceramic masses are also considered suitable candidates for scaffolds generation due to their surface reactive coatings, which ensure the bioactivity of these types of structures [8,19].

Nevertheless, composite materials obtained by integrating at least two different types of materials, such as ceramics, metals or polymeric structures, are considered to be the most optimized strategy for the scaffolding process [14], ensuring superior features, especially related to the mechanical strength and osteoconduction process of the scaffold-type structure [18]. It is also important to be mentioned that ceramic masses have already showed encouraging clinical results in maxillary sinus augmentation procedures [20,21]. In addition to this, digital dentistry approaches and microdiagnostics provide a more accurate planning procedure, resulting in an improved surgical outcome [22,23,24].

In the present study, the foam replication method was employed to produce two scaffold-type structures using different Ceramco iC biomaterials. However, akin to any newly developed structure, the generated samples possess different features compared to the raw materials; thus, concerns about their clinical value arose, causing the need for physicochemical assessments and primary biological screening.

In addition to the synthesis and physicochemical evaluation of ceramic scaffolds, a second aim of the current study was to compare the newly synthetized scaffold ceramics with two classical augmentation products (Cerabone^®^) in terms of morphological aspects, especially the porous network of structures, but also the elemental composition, and to further correlate these aspects with the biological response by testing the all four ceramic samples through conventional in vitro and in ovo assays, which have already been successfully employed to establish the biological profiles of different dental materials [25,26,27]. Thus, as an in vitro model, primary human gingival fibroblasts (HFG cells) were used, while in ovo screening was performed by means of chick chorioallantoic membrane (CAM) test to determine the possible irritating impact of the samples on the vascular plexus.

## 2. Materials and Methods

### 2.1. Reagents

In the current study, four types of ceramic samples used for bone augmentation were assessed: the first two samples were made out of Cerabone Granules (Botiss Biomaterials GmbH, Zossen, Germany), a well-known material dedicated for bone augmentation—P1 = Cerabone^®^ Granulate, size 0.5–1.0 mm; P2 = Cerabone^®^ Granulate, size 1.0–2.0 mm. In parallel, two kinds of commercial ceramic masses were used to develop two scaffold samples (P3 and P4). The P3 sample was obtained from Ceramco iC Natural Dentine, Dentsply Sirona, whereas the P4 sample was synthesized from Ceramco iC Natural Enamel, Dentsply Sirona. Both types of Ceramco iC Natural samples are usually used for dental prostheses, such as crowns and bridges, as well as facets.

The ceramic masses exhibit the following physical properties: CTE 12.0 × 10^−6^ K^−1^ (20–500 °C); for porcelain-bonding alloys in the CTE range, 13.8–15.1 × 10^−6^ K^−1^ (20–500 °C); firing temperature, 840 °C (1st Dentin); heating rate, 100 °C/min; flexural strength, 90 N/mm^2^; leucite grain size, 1–5 μm. Regarding their composition, the two Ceramco iC masses are made up of 80–100% sodium potassium aluminosilicate and 0–5% tin oxide.

### 2.2. Synthesis of the Scaffold-Type Structures (P3, P4)

**Figure 1:**
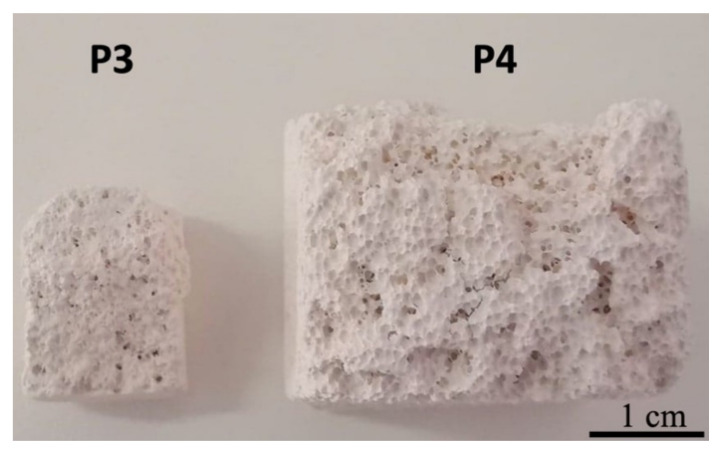
General overview of the ceramic scaffolds P3 and P4. Scale bar represents 1 cm.

The scaffold-based samples were realized by the foam replication method [28], which involves the production of ceramic foams by coating a polymeric sponge, used as an organic template, with a ceramic slurry. Afterwards, the sponge was burned out during a proper heat treatment which, at the same time, sinters the ceramic powders. Two types of polyurethane sponge were used to obtain the scaffold-types samples: the green sponge, which presents a density of 300 kg/m^3^, was used to ensure a larger density structure with smaller and fewer pores, which led to generation of the P3 sample, while the blue sponge, with a density of 210 kg/m^3^, was used to generate larger pores and consequently a more porous structure, thus obtaining the P4 sample. The slurries were prepared by dispersing the porcelain powder into distilled water together with a polyvinyl binder under magnetic stirring. The binder was used to control both the slurry viscosity and the ability of the solid particles to coat the sponge. Then, the sponges, cut to the wanted shape, were manually immersed in the slurry and, unlike the traditional replication method, they were not squeezed but kept fully loaded with the slurry. The sponges were immediately dried under a multidirectional air flux at 120 °C for 30 min. The postforming thermal treatments were performed in an electric furnace at 1000 °C for 120 min (Nabertherm L15/12/P330 Muffle Furnace, Nabertherm GmbH, Lilienthal, Germany) to burn out the organic phase and sinter the ceramic structure. After drying, the polymer foam was burned out slowly at 420 °C to avoid damaging the ceramic body. Once the polymer sacrificial template was removed, the samples were sintered to the desired structure using a predetermined and optimized heat-treatment schedule. An overview of the newly generated ceramic scaffold-type structures is depicted in Figure 1.

### 2.3. SEM–EDAX Analysis

In scanning electron microscopy (SEM) imaging analyses, the Quanta 250 FEI microscope (Eindhoven, The Netherlands) was employed using different magnifications in order to allow both the overall analysis and the ultrastructure details. To collect the data by SEM analyses the following procedures were employed: (i) BSE/backscattered electrons were used for topography analyses; (ii) EDX analysis (energy dispersive X-ray spectroscopy) with an EDAX system (Apollo X detector, Ametek, Mahwah, NJ, USA). The samples were covered with gold, 4 nm/deposition, 3 times, using an AutoAgar Sputter Coater (Agar Scientific Ltd., Essex, UK).

The software Scandium 5.0 (Olympus Soft Imaging Systems, Münster, Germany) were used for measurements, while the SmartSCAN™ scan strategy and Drift Compensated Frame Imaging (DCFI) for video acquisition. Both softwares are incapsulated in the server platform of the SEM Quanta 250 microscope (FEI Europe, The Netherlands).

The number of measurements (n) performed to determine dimension of the pores were made at the same order of magnification (2000×): for small pores n = 30; for large pores n = 10. The pores were randomly chosen.

### 2.4. Artificial Saliva

The artificial saliva was prepared as a modification of Meyer’s solution as described by [29] by obtaining a solution with a pH of 7.355. Afterwards, to obtain different pH values of saliva, the pH was modified as follows: (i) for alkaline saliva—addition of NaOH 10N until a pH of 10.769 was achieved; (ii) for acid saliva—addition of HCl until the artificial saliva reached the pH value of 3.393 [30]. All three types of artificial saliva were used to immerse the ceramic samples for 8 days and to analyze possible morphological alterations that may occur as a result of this experimental setup. The pH values were determined with maximum measurement reliability by using a Schott pH-meter (Schott Instruments GmbH, Mainz, Germany).

### 2.5. X-ray Fluorescence (XRF) Analysis

A wavelength dispersive X-ray fluorescence (X-MET8000 series, HHXRF models) spectrometer (Hitachi, Chiyoda, Japan) was used to conduct experiments on ceramic samples at Spectroscopy Laboratory Banat University of Agricultural Sciences and Veterinary Medicine “King Mihai I of Romania” in Timisoara. The calibration of the fundamental parameters of the X-MET8000 (FP) was optimized for the analysis of the ceramic matrices. It is based on the principles of physics to calculate the elementary composition of the measured sample. The accuracy of the measurements was verified by assessing the samples, for which the concentration of each mixture or alloy are indicated on the sample or in the kit tables. The samples were air dried for 48 h, ground and sieved using 0.1 mm mesh size sieve to have uniform particle size. Each sample was labelled and stored in a dry plastic container that had been precleaned with concentrated nitric acid prior to analysis with an X-ray fluorescence (XRF) spectrometer. Samples were measured with the portable stand for complete protection of the user against scattered radiation, according to the literature [31,32,33].

### 2.6. In Vitro Experimental Setup

To evaluate the in vitro impact of ceramic samples on primary human gingival fibroblasts (HGFs), the test samples (P1, P2, P3, P4) were prepared according to the protocol described by Thrivikraman et al. [34]. Thus, the bulk sample materials were hand grinded using mortar and pestle until an ultrafine powder was obtained for each sample. Thereafter, to obtain the stock solutions, ultrafine powders of the four test ceramics were suspended in cell culture media at a concentration of 2 mg/mL. The stock solutions were further used to obtain the final test concentrations (5, 10, 25 and 75 µg/mL), which were used to stimulate the HGF cells for the evaluation of the cell viability.

### 2.7. Cell Line and Cell Culture Conditions

Primary human gingival fibroblasts—HGF cells were used in the present study to perform the in vitro testing of the sample dental ceramics. The cell line was purchased from American Type Culture Collection (ATCC, code no PCS­201­018™, LGC Standards GmbH, Wesel, Germany). The culture conditions for this cell line consists of Fibroblast Basal Medium (ATCC^®^ PCS-201-030™), supplemented with Fibroblast Growth Kit-Low serum (ATCC^®^ PCS-201-041™) and 0.1% Penicillin-Streptomycin-Amphotericin B Solution (ATCC^®^ PCS-999-002™). All in vitro procedures were performed under standard conditions: the cells were maintained in humidified atmosphere containing 5% CO_2_ and 37 °C in a Steri-Cycle i160 incubator (Thermo Fisher Scientific, Inc., Waltham, MA, USA). The in vitro experiments were performed under sterile conditions using a biosafety cabinet—MSC Advantage 12 model (Thermo Fisher Scientific, Inc., Waltham, MA, USA).

### 2.8. Cell Viability Assay by Means of Alamar Blue Test

The effect induced by test ceramics at different concentrations (5, 10, 25, 75 µg/mL) on the primary human gingival fibroblasts (HGFs) was determined by applying the Alamar blue colorimetric test, as previously described in detail by our group [35,36]. In brief, a density of 10^4^ cells/well were cultured onto 96-well plates and incubated overnight. The next day, the old medium was removed and the cells were stimulated with four concentrations (5, 10, 25, 75 µg/mL) of dental ceramic ultrafine particle suspension for different intervals of time (24, 48 and 96 h). The control cells were maintained in the same conditions as the stimulated ones; however, they were treated with specific culture medium.

At the end of the stimulation period, 20 µL/well of Alamar blue reagent was added at a concentration of 0.01% for a period of at least 3h. During this time, the cell plates were maintained under standard conditions (humidified atmosphere with 5% CO_2_ and 37 °C) in the Steri-Cycle i160 incubator (Thermo Fisher Scientific, Waltham, MA, USA).

To quantify the cell viability percentage, the absorbance of each well was determined spectrophotometrically at two wavelengths (570 and 600 nm) by means of a microplate reader (xMark^TM^ Microplate, Bio-Rad Laboratories, Hercules, CA, USA) and the following formula was applied [37]:

$$Cell\;viability\;\left(\%\right)=\frac{(\varepsilon_{OX})\lambda_{2}\;\cdot A_{test}\;\lambda_{1}-\left(\varepsilon_{OX}\right)\lambda_{1}\cdot A_{test}\;\lambda_{2}}{\left(\varepsilon_{OX}\right)\lambda_{2}\cdot A_{0}\lambda_{1}-\left(\varepsilon_{OX}\right)\lambda_{1}\cdot A_{0}\lambda_{2}}$$

Legend:

*ε_ox_* = molar extinction coefficient of the oxidized form of Alamar blue reagent;
*A_test_* = absorbance of test wells;
*A*_0_ = absorbance of control well;
*λ*_1_ = 570 nm;
*λ*_2_ = 600 nm.

### 2.9. HET–CAM Method

For the evaluation of the possible irritative effect induced by test dental ceramics, the HET–CAM test was performed, respecting the guideline of the Interagency Coordinating Committee on the Validation of Alternative Methods (ICCVAM) [38]. In brief, fertilized eggs from Gallus gallus domesticus were sprayed with ethanol (70%) to ensure disinfection of the eggshell and afterwards the eggs were placed in an incubator at 37 °C and high humidity. Further, a slightly modified protocol developed by our group was employed [25,39].

The experimental procedure was conducted on day 9 of embryonic development. Test samples next to positive control (SLS, sodium lauryl sulphate 0.5% in distilled water) and negative control (culture media) were inoculated on the developing CAM at a volume of 300 µL. The evaluation was performed under a stereomicroscope (Discovery 8 Stereomicroscope, Zeiss, Göttingen, Germany). Relevant images before application (t_0_) and 300 s after inoculation (t_5_) were captured (Axio CAM 105 color, Zeiss, Göttingen, Germany) and processed using Zeiss ZEN software Carl Zeiss Microscopy GmbH, Göttingen, Germany; https://www.zeiss.com/microscopy/int/products/microscope-software.html, Gimp 2.8 and ImageJ software (v 1.50e, U.S. National Institutes of Health, Bethesda, MD, USA).

The evaluation consisted of monitoring for 300 s the live modifications observed on the vascular bed of the CAM after sample application in terms of hemorrhage—H (blood vessel bleeding), vascular lysis—L (disintegration of blood vessels), coagulation—C (intra- or extravascular protein denaturizing). For each parameter, the moment (in seconds) when the first event appeared was noted. Subsequently, an irritation score (IS) was calculated using the following formula:

$$\mathrm{IS}=5\times \frac{301-\mathrm{Sec}\;H}{300}+7\times \frac{301-\mathrm{Sec}\;L}{300}+9\times \frac{301-\mathrm{Sec}\;C}{300}$$

where: H = hemorrhage; L = vessel lysis; C = coagulation; hemorrhage time (Sec H) = onset of hemorrhage reactions on CAM (in seconds); lysis time (Sec L) = onset of vessel lysis on CAM (in seconds); coagulation time (Sec C) = onset of coagulation formation on CAM (in seconds).

Means values were obtained. The IS values range on a scale between 0 and 21, and the irritation potential of the tested samples was described according to a scale set by Luepke: 0–0.9—nonirritant, 1–4.9—weak irritant, 5–8.9/9.9—moderate irritant, 8.9/9.9–21—strong irritant [40].

### 2.10. Statistical Analysis

The GraphPad Prism 5 software (GraphPad Software, San Diego, CA, USA) was employed to perform and present the statistical analysis data. A one-way ANOVA test was applied to determine the statistical differences of test samples versus control, followed by Tukey’s post-test (* *p* < 0.05, ** *p* < 0.01, and *** *p* < 0.001).

## 3. Results

### 3.1. SEM–EDAX Analysis

Since one of the most essential features of a ceramic material used for bone augmentation is related to its network porosity, the morphological analysis of the samples was a mandatory analysis of the present study. In this regard, the surface topography and the ultrastructure details together with the chemical species of the dental ceramic samples were determined through SEM–EDAX analysis.

**Figure 2:**
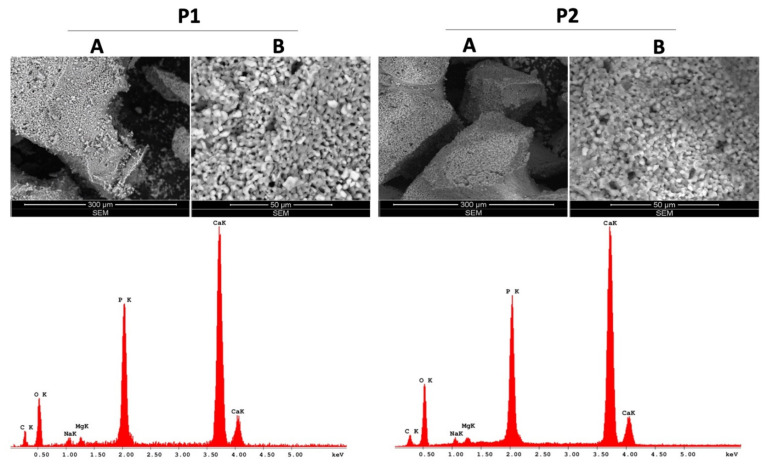
SEM–EDAX analysis of the traditional Cerabone^®^ ceramics (P1, P2); **A**—represents the general aspects of the samples and **B**—represents the ultrastructure details of the samples.

As presented in Figure 2, SEM micrographs of Cerabone^®^ samples exhibit similarities in terms of overall surface topography. Additionally, EDAX analysis revealed the same chemical species in the case of both traditional ceramics (P1 and P2).

**Figure 3:**
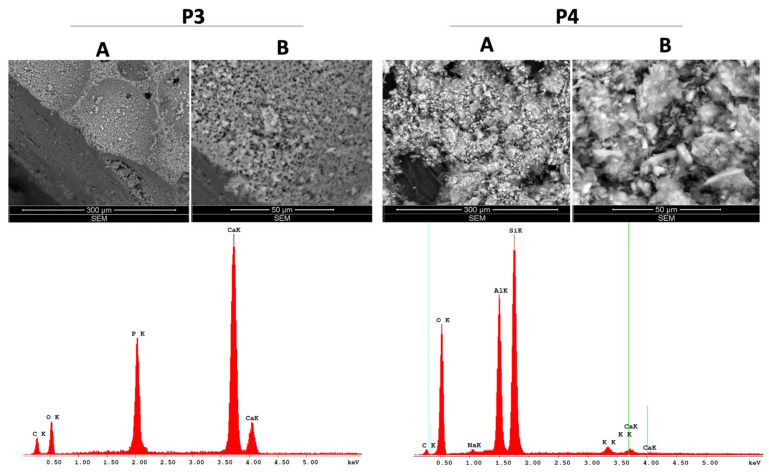
SEM–EDAX analysis of the scaffold-type ceramics (P3, P4); **A**—represents the general aspects of the samples and **B**—represents the ultrastructure details of the samples.

In both scaffold-type samples, P3 and P4 (Figure 3), two distinct regions are encountered: the median region presents a relatively uniform pore distribution with high dimensional scattering, whereas the peripheral region has a lower porosity and higher compactness, simulating the natural bone porosity. However, a “rocky”, microflake surface appearance was observed in P4 with a noneasy visible pore material.

In addition, the overall surface topography of all ceramic samples was evaluated at different pH values, after maintaining the samples into three types of artificial saliva (acidic, neutral and alkaline) for 8 days, at 37 °C and 250 RPM using an on–off shaking procedure. The results revealed that only the alkaline saliva induces several alterations of the morphological aspect of ceramic samples, as several depositions could be observed under these conditions (Figure S3), whereas the neutral and acidic pH did not induce significant changes in terms of morphology (Figures S1 and S2).

Following the SEM analysis, two different classes of pores were identified in terms of their dimensions. The values of the pores measurements are summarized in Table 1.

The chemical species of the test ceramics were identified through EDAX analysis and are presented in detailed as weight percent (Wt %) and atomic percent (At %) in Table 2.

As presented in Table 1, the elemental compositions of P1 and P2 samples are quite similar, with P1 presenting higher weight percentages of C, Na and Mg compared to P2: 17.38%, 2.20% and 1.45% versus 12.77%, 1.38% and 1.19%, while P2 has a higher amount of O; the percentages of P and Ca are very similar between P1 and P2 and represent over 40% of the weight of these samples. However, the elemental composition of P3 is the simplest of the four dental ceramic samples tested, as 64.92% of its composition is based on two elements, Ca and O, while 35.08% is represented by C (21.71%) and P (13.37%). Nevertheless, the structure of P4 is the most different in terms of elemental composition, as it is not based mainly on Ca, as was the case of the above-mentioned samples (P1, P2, P3). In this particular case, the most significant share (over 89%) is O (45.48%), Si (28.36%) and Al (15.59%), while Ca represents only 0.56% of the P4 sample weight.

### 3.2. Analysis of the Content of Trace Metals in Ceramic Samples

To evaluate the metal traces present in the ceramic scaffold samples (P3 and P4) comparative to the classical augmentation ceramic masses (P1 and P2), X-ray fluorescence (XRF) analysis was employed and the results are expressed in ppm, within Table 3.

The results obtained through XRF analysis revealed that none of the ceramic samples possess traces of metals that could be considered noxious for the clinical use of the test structures. In addition, the quantification of the chemical elements recorded by this method correlates with the elemental analysis obtained through the EDAX analysis.

### 3.3. Cell Viability Assessment

**Figure 4:**
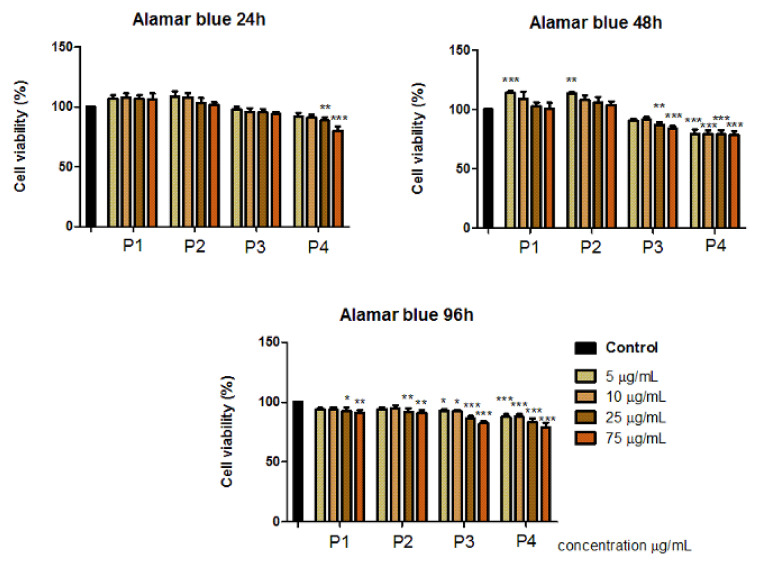
Primary Human Gingival Fibroblast (HGF) response to dental ceramic specimens after 24, 48 and 96 h. The results are presented as cell viability percentage (%) normalized to control cells. The graph bars are expressed as mean values ± SD of three independent experiments. One-way analysis of variance (ANOVA) test was performed to determine the statistical differences of test sample versus control, followed by Tukey’s multiple comparisons post-test (* *p* < 0.05, ** *p* < 0.01, *** *p* < 0.001).

To evaluate the effect induced by dental ceramic specimens (P1, P2, P3, P4) on primary human gingival fibroblasts (HGFs), the Alamar blue proliferation test was performed at three different intervals of time (24, 48, 96 h) and the results obtained are presented within Figure 4.

The results indicate that the primary human gingival fibroblasts (HGFs) manifested no significant cytotoxic effects after treatment with the test dental ceramics (P1, P2, P3, P4) at concentrations of 5, 10, 25 and 75 µg/mL, following 24, 48 and 72 h exposure.

P1 and P2 induced a proliferative activity (viability above 100%) of cell population when applied at concentrations of 5 and 10 µg/mL, this effect was especially observed after 48 h. However, after exposure to the highest test concentration, 75 µg/mL, the viable rate of HGF cells was around 90%.

After treatment with P3 and P4 samples, HGF cells manifested lower cell viability percentages. In this case, the most decreased cell viability rates were recorded after 48 h, following exposure to the highest concentration of 75 µg/mL, the cells manifested a viability rate of 84.12 ± 1.74% after stimulation with P3, respectively, a viability of 79.15 ± 3.50% after treatment with P4.

### 3.4. Irritation Potential Assessment by the Means of HET–CAM Test

**Figure 5:**
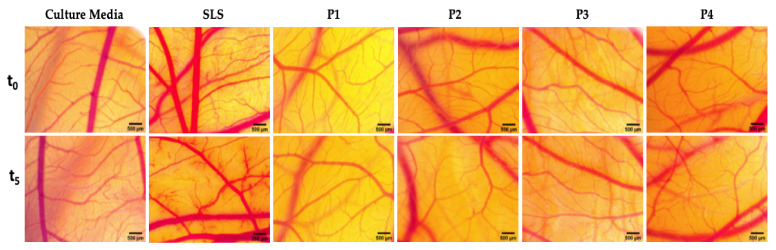
Stereomicroscope images of the CAMs inoculated with controls (culture media used as negative control and sodium lauryl sulfate—SLS 0.5% was used as positive control) and test dental ceramics (P1, P2, P3, P4). The pictures were taken before the application (t_0_) and after 5 min of contact with the test compounds (t_5_). Scale bars represent 500 µm.

A possible toxicological effect of the dental ceramic specimens was also evaluated in vivo by employing the in ovo chick chorioallantoic membrane (CAM) assay. This technique permits the evaluation of the irritant potential of test ceramics in a biological environment (Figure 5).

As depicted in Figure 5, the four dental ceramics induced no vascular toxicity (hemorrhage, coagulation or vessel lysis) on the vascular developing CAM after 5 min of exposure. This response is comparable with the one induced by the negative control (culture media). In contrast, the positive control (SLS 0.5%) induced sever irritative events.

All the tested ceramics can be classified as nonirritants (Table 4). The only ceramic material that induced some modifications upon microvessel circulation was P2, characterized by a higher granulation. Still, the impact on the affected parameters was low, and the overall effect did not induce an irritative phenomena. All tested ceramic materials are nonirritative and did not induce any toxic reaction on the chorioallantoic membrane of the chick embryo.

## 4. Discussion

The current study presents the development of two scaffold-type structures (P3 and P4), obtained by the foam replication method, by using a polymeric sponge that acts as a template for porous scaffolds—a technique that has gained widespread popularity among all the other scaffolding processes due to good thermal and chemical stability, but also due to the high interconnected network of the resulting structure [41]. Moreover, by employing this method, solid polymers with a precise replica of the geometric characteristics of the polymeric sponge can be obtained and since the so-called template sponge can be hand-crafted according to specific bone defects, the method can produce personalized scaffolds for individual patients’ needs [3]. In addition, the method offers the advantage of choosing between various polymeric sponges that are characterized by different densities, a parameter that plays a key role in obtaining tailored porosity of the final scaffold structure. Thus, the lower the density of the sponge, the more it can generate the formation of porous network with high pore interconnectivity. This aspect plays an even more important role as the porosity of the ceramic scaffolds can be considered one of the most important features of these structures, as it orchestrates the formation of an optimal microenvironment between the scaffold and the surrounding tissue by forming chemical bonds with the cells, as well as promoting the osteoconduction process by facilitating cell migration at the bone tissue site [4,17,42]. Due to the above-mentioned features, the scaffold-type structures could also be considered as good candidates for bone grafting materials for patients suffering from systemic disease, in which case the implant rehabilitation process is more sensitive [43].

Since, the bone microstructure is characterized by a gradient of porosity, which varies depending on the transversal section of the bone (the size of the pores decreases the closer they are to the surface of the bone structure) [44], ceramic scaffolds are considered a suitable approach for bone tissue restoration as they are characterized by different pore sizes, thus ensuring complex biological functions, as follows: (i) nanosized pores provide an optimal surface for protein and cell attachment; (ii) pores up to 100 μm offer the possibility of capillary development, nutrient flux and waste product excretion; and (iii) pores larger than 150 μm and up to 800 μm facilitate bone tissue ingrowth and the formation of blood vessels [15,16], and once the vascular supply becomes functional, it further provides the growth factors necessary for the cell proliferation, thus ensuring the generation of the osteoinduction process [4]. Nevertheless, an optimal balance between the porosity of the scaffold and its mechanical features should be acquired to obtain a suitable structure with good clinical outcome [3,10].

The characterization of the scaffold-type samples (P3 and P4) through SEM analysis (Figure 3) revealed that the pore morphology is an open one, comprising pores with variable dimensions depending on the area of analysis; when analyzing the internal surface, the pores are more numerous, comprising both micropores and macropores (Table 1), whereas the external structure of the scaffolds mimics cortical bone tissue, exhibiting a higher density and reduced porosity—as mentioned in the literature as being optimal for bone augmentation [44]. In other words, the resulting samples have an original structure that fits with a resilient outer surface and a very porous internal network. In particular, the outer surface behaves as a “shell” and guarantees both high permeability and maneuverability.

Altogether, it can be stated that the replication method employed for the development of the both scaffold-type structures led to the generation of two structures that reveal similar features—gradient porosity—of natural bone.

On the other hand, the present study also focuses on comparing the newly developed 3D scaffolds (P3 and P4) with the two traditional Cerabone^®^ structures (P1 and P2), both in terms of physico-chemical features and biological profile.

Cerabone^®^ is a 100% pure bone mineral of bovine origin manufactured by a unique 1200 °C production process [45] and it has been considered the most widely use and successful bone replacement material [46], being applied in more than 1 million patients in regenerative dentistry and has already been in use for more than 15 years in various medical applications (e.g., craniofacial surgery, oncology and hand and spine surgery). The sophisticated processing of the bovine bone removes all organic components, resulting in a bone mineral with exceptional purity. In addition, potential infectious agents such as bacteria, viruses and prions are removed through the high temperature treatment [45]. Both product and production process are fulfilling applicable international regulatory and safety requirements for xenogeneic bovine bone grafting materials in agreement with International Standards referring to medical devices that use animal tissues and derivatives, regarding (i) risk management—ISO 22422-1:2020 [47] (ii) controls on sourcing, collection and handling ISO 22442-2:2020 [48] and (iii) elimination/inactivation of viruses and transmissible spongiform encephalopathy (TSE) agents ISO 22442-3:2007 [49].

The SEM analysis of the classical augmentation masses (P1 and P2) showed a low porosity grade with few nanopores, indicating a lower simulation of the bone structure, an aspect that may be caused by the high-temperature setup of the synthesis protocol employed to obtain Cerabone^®^ granules [50].

In addition, the SEM results obtained for all ceramic samples after immersion in three types of artificial saliva (alkaline, neutral and acidic saliva, depending on the pH values) revealed no significant changes in terms of morphological aspects when neutral and acidic artificial saliva were used (Figures S1 and S2). However, a slight alteration of samples morphology was observed when alkaline saliva was used, several deposits being identified on the samples’ surface (Figure S3). Thus, based on these results, a high-alkaline medium should be avoided in clinical practice when ceramics are used as dental bone substitutes.

The biological impact induced by bone grafting materials are of major importance for a successful regenerative outcome. One of the key aspects that should be developed by the cells surrounding the bone implant is related to the vascularization efficacy of the implanted material [51]. In this regard, bone marrow stem cell (BMSC) and endothelial cell cocultures have been shown to enhance the development of new vessels when employed in a procedure for calvaria bone repair [52]. Based on these data, the biological evaluation of graft materials should be especially focused on these two types of cells.

Nevertheless, as an alternative in vitro model, human dental pulp stem cells (HDPSCs) and human gingival fibroblasts (HGFs) can be employed to assess the biological profile of regenerative materials, as these type of cells manifest similar biological behaviors to that of BMSCs [53]. Additionally, the literature is scarce of data related to the biological impact of dental ceramics at the gingival and periodontal levels in terms of inflammatory activity [53].

Regarding the cellular-related aspects mentioned above, the biocompatibility of the newly synthesized scaffold structures (P3 and P4) was assessed on primary human gingival fibroblasts (HGF cells) and the results are presented in comparison with those of the classical ceramic samples (P1 and P2). As shown in Figure 4, distinct results of the viable cell population were observed. As the main difference, Cerabone^®^ samples (P1 and P2) induced proliferation of HGF cells especially after 48 h, following exposure to low concentrations (5 and 10 µg/mL). This aspect was somehow expected as Cerabone^®^ granules have already been demonstrated to facilitate the proliferation of osteoblasts after a short time post-implantation due to the hydrophilic structure of the material [45].

Another different response that was observed after treatment of HGF cells with P3 and P4 samples, compared to samples P1 and P2, is that the viability of gingival fibroblasts was more affected after stimulation with concentrations of 25 and 75 µg/mL of the scaffold samples (P3 and P4), the cell viability recorded in these cases being about 78%, whereas gingival fibroblasts treated with the same concentrations of traditional ceramic samples (P1 and P2) induced only a slight decrease in HGF cell population, showing a cell viability rate over 90%.

Nevertheless, none of the tested samples can be labeled as cytotoxic as, according to ISO Standard 10993-5:2009 regarding Biological Evaluation of Medical Devices [54], one compound is classified as cytotoxic only if the viability of the treated cells has been affected by more than 30%.

In addition, the four ceramic materials were examined in vivo using the HET–CAM assay in order to establish their irritative potential upon mucosal highly vascularized tissues. All tested materials were evaluated as safe, with no irritation risk associated upon mucosal application. Firstly, used as an alternative to the Draize eye test, the HET–CAM protocol is a simple, cheaper and less time-consuming alternative, suitable for assessing irritative and inflammatory potential of various types of chemicals with topical application [55,56]. Recent studies in the field of nanomaterials and other types of dental materials explore the biocompatibility of such products using this in vivo technique [27,57]. Additionally, metal alloys were studied and their bioavailability could be established by using the HET–CAM assay, with the possibility of analyzing the impact of certain metals released in the tested media [58]. In this regard, our research group recently demonstrated the lack of irritative potential and completed the biocompatibility data of dental materials such as mini-implants [25,39], thus confirming the benefits of this assay in toxicity studies for materials with topical application including dental use.

Summarizing all the biological effects, induced by the four ceramic samples (P1, P2, P3 and P4), on HGF cells and also on the chorioallantoic membrane of the chick embryos, the present data indicated that the test specimens are biocompatible materials, thus providing reliable results for the possible upcoming dental applications of the newly developed ceramic scaffolds.

Nevertheless, for a more complex in vitro biological profile that could provide additional data regarding the vascularization process that may be encountered in vivo after the implantation process, the ceramic scaffolds should be evaluated using an in vitro model based on endothelial cells, together with the CAM assay employed to evaluate the angiogenesis process.

## 5. Conclusions

The proposed materials (P3 and P4) have developed comparable properties with standard ones (P1 and P2), revealing good mineral properties and suitable compositions for biological use. Moreover, these products seems to be highly compatible with the biological microenvironment. The data are supported by their biocompatible and low toxic behaviours developed on the basis of in vitro experimental evaluations and also after employing in ovo CAM assay as an additional observation of their impact on blood vessels level. The scaffold-type ceramics are nontoxic to primary sensitive cells, such as gingival fibroblasts, and are also considered as stable structures under various biologic conditions, as changing the pH of saliva did not significantly alter their morphological aspects. Nevertheless, novel directions on the synthesis and development of ceramic scaffolds are helpful in the use of these biocompatible products as high-impact tools for practical applications in dentistry. Fast, applicable and reproducible testing is mandatory to confirm their safety and biological approvement.

## Figures and Tables

**Table 1:** Pore size measurements.

Sample *	Large Pore (Mean ± SD)	Small Pore (Mean ± SD)
P1	8.302 ± 0.763	1.374 ± 0.267
P2	5.209 ± 0.724	1.334 ± 0.236
P3	6.138 ± 1.441	0.921 ± 0.238
P4	29.590 ± 3.563	2.309 ± 0.493

* All measurements values are expressed in μm. The pores were randomly chosen. Number of pore size measurement determinations (n): for small pores n = 30; for large pores n = 10.

**Table 2:** Elemental composition of test ceramics.

Sample	Element	Wt %	At %	K-Ratio	Z	A	F
	C k	17.38	28.45	0.0358	1.0404	0.1980	1.0005
	O k	37.39	45.95	0.0474	1.0246	0.1273	1.0002
	Na k	2.20	1.88	0.0047	0.9609	0.2199	1.0018
P1	Mg k	1.45	1.17	0.0043	0.9857	0.3025	1.0033
	P k	14.79	9.39	0.0987	0.9536	0.6952	1.0071
	Ca k	26.80	13.15	0.2386	0.9578	0.9296	1.0000
	Total	100.00	100.00				
	C k	12.77	21.15	0.0262	1.0403	0.1972	1.0006
	O k	43.57	54.15	0.0587	1.0244	0.1316	1.0002
	Na k	1.38	1.19	0.0028	0.9608	0.2112	1.0018
P2	Mg k	1.19	0.97	0.0035	0.9855	0.2961	1.0033
	P k	14.76	9.48	0.0977	0.9535	0.6896	1.0070
	Ca k	26.32	13.06	0.2341	0.9577	0.9286	1.0000
	Total	100.00	100.00				
	C k	21.71	35.36	0.0498	1.0395	0.2207	1.0005
P3	O k	33.37	40.80	0.0378	1.0236	0.1107	1.0001
	P k	13.37	8.44	0.0937	0.9527	0.7288	1.0089
	Ca k	31.55	15.40	0.2867	0.9571	0.9496	1.0000
	Total	100.00	100.00				
	C k	8.64	13.81	0.0113	1.0367	0.1256	1.0005
	O k	45.48	54.58	0.1118	1.0210	0.2407	1.0005
	Na k	0.56	0.47	0.0015	0.9576	0.2725	1.0050
P4	Al k	15.59	11.10	0.0808	0.9540	0.5375	1.0103
	Si k	28.36	19.39	0.1280	0.9824	0.4594	1.0002
	K k	0.81	0.40	0.0057	0.9297	0.7537	1.0008
	Ca k	0.56	0.27	0.0044	0.9533	0.8204	1.0000
	Total	100.00	100.00				

**Table 3:** Metals composition of ceramics (ppm).

Ceramic Sample		Trace Metals Quantification				
P1	Mg	Al	Si	P	Ca	V
	1.5698	0.0463	0.925	6.8278	48.8914	0.4923
	Cr	Mn	Fe	Co	Ni	Cu
	0.0536	0.0146	0.079	0.0104	0.0084	0.0043
	Zn	Sr	Zr	Mo	Ag	Cd
	0.0502	0.1075	0.002	0.0025	0.0122	0.0089
	Sn	Sb	Ba	Au	Hg	Pb
	0.0216	0.0088	0.0837	0.0063	0.0016	0.0012
	Th	K	PD			
	0.0016	0	0			
P2	Mg	Al	Si	P	Ca	V
	1.3591	0	0.8319	7.3158	48.4828	0.4583
	Cr	Mn	Fe	Co	Ni	Cu
	0.0992	0.0368	0.0883	0	0	0
	Zn	Sr	Zr	Mo	Ag	Cd
	0.0437	0.0954	0.0018	0.0023	0.0141	0.0086
	Sn	Sb	Ba	Au	Hg	Pb
	0	0.0085	0.045	0.0073	0	0
	Th	K	PD			
	0.0017	0	0			
P3	Mg	Al	Si	P	Ca	V
	0.2379	0	1.6962	6.9812	46.0344	1.939
	Cr	Mn	Fe	Co	Ni	Cu
	0.1782	0.092	0.2095	0.0234	0.0203	0.0022
	Zn	Sr	Zr	Mo	Ag	Cd
	0.0101	0.0381	0.0021	0.0061	0.0241	0.0327
	Sn	Sb	Ba	Au	Hg	Pb
	0.0719	0.0566	0.141	0.0147	0.0024	0.0038
	Th	K	PD			
	0.0031	0.6141	0.5678			
P4	Al	Sc	K	Ca	V	As
	2.710	0.0039	0.6522	0.34443	0.0007	0.0004
	Fe	Co	Zn	Rb	Sr	Zr
	0.200	0.0001	0.0059	0.0055	0.0039	0.0048
	Ti	U	Ta	Pb	Th	
	0.0348	0	0.002	0.003	0.0015	

**Table 4:** Classification of the irritant potential of dental ceramic specimens (P1, P2, P3, P4) and controls.

Test Compounds & Controls	Irritation Score	Classification of the Effect
Culture media (Negative control)	0	Nonirritant
SLS 0.5% (Positive control)	13.45	Strong irritant
P1	0	Nonirritant
P2	0.81	Nonirritant
P3	0	Nonirritant
P4	0	Nonirritant

## Data Availability

Data sharing is not applicable for this article.

## Supplementary Materials

The following are available online at https://www.mdpi.com/article/10.3390/ma14092266/s1, Figure S1: SEM micrographs of the ceramic samples (P1, P2, P3, P4) after 8 days immersion in acidic saliva (pH = 3.393) at 37 °C and 260 RPM with on-off shaking protocol; A–represents the general aspects of the samples and B–represents the ultrastructure details of the samples; Figure S2: SEM micrographs of the ceramic samples (P1, P2, P3, P4) after 8 days immersion in neutral saliva (pH = 7.355) at 37 °C and 260 RPM with on-off shaking protocol; A–represents the general aspects of the samples and B–represents the ultrastructure details of the samples; Figure S3: SEM micrographs of the ceramic samples (P1, P2, P3, P4) after 8 days immersion in alkaline saliva (pH = 10.769) at 37 °C and 260 RPM with on-off shaking protocol; A–represents the general aspects of the samples and B–represents the ultrastructure details of the samples.

## References

[1] Babu P.J., Alla R.K., Alluri V.R., Datla S.R., Konakanchi A. (2015). Dental Ceramics: Part I-an overview of composition, structure and properties. Am. J. Mater. Eng. Technol..

[2] Trajkovski B., Jaunich M., Müller W.-D., Beuer F., Zafiropoulos G.-G., Houshmand A. (2018). Hydrophilicity, Viscoelastic, and Physicochemical Properties Variations in Dental Bone Grafting Substitutes. Materials.

[3] Baino F., Novajro G., Vitale-Brovarone C. (2015). Bioceramics and Scaffolds: A Winning Combination for Tissue Engineering. Front. Bioeng. Biotechnol..

[4] Bernardi S., Macchiarelli G., Bianchi S. (2020). Autologous Materials in Regenerative Dentistry: Harvested Bone, Platelet Concentrates and Dentin Derivates. Molecules.

[5] Pizzicannella J., Pierdomenico S.D., Piattelli A., Varvara G., Fonticoli L., Trubiani O., Diomede F. (2019). 3D Human Periodontal Stem Cells and Endothelial Cells Promote Bone Development in Bovine Pericardium-Based Tissue Biomaterial. Materials.

[6] Pollington S., van Noort R. (2009). An Update of Ceramics in Dentistry. Int. J. Clin. Dent..

[7] Ho G.W., Matinlinna J.P. (2011). Insights on Ceramics as Dental Materials. Part I: Ceramic Material Types in Dentistry. Silicon.

[8] Kumar P., Kumar V., Kumar R., Kumar R., Pruncu C.I. (2020). Fabrication and characterization of ZrO_2_ incorporated SiO_2_–CaO–P_2_O_5_ bioactive glass scaffolds. J. Mech. Behav. Biomed. Mater..

[9] Schitea R.-I., Nitu A., Ciobota A.-A., Munteanu A.-L., David I.-M., Miu D., Raileanu M., Bacalum M., Busuioc C. (2020). Pulsed Laser Deposition Derived Bioactive Glass-Ceramic Coatings for Enhancing the Biocompatibility of Scaffolding Materials. Materials.

[10] Yamada M., Egusa H. (2017). Current bone substitutes for implant dentistry. J. Prosthodont. Res..

[11] Saima S., Jan S.M., Shah A.F., Yousuf A., Batra M. (2016). Bone grafts and bone substitutes in dentistry. J. Oral Res. Rev..

[12] Gabor A., Hosszu T., Zaharia C., Kozma A., Cojocariu A.C., Negrutiu M.L., Szuhanek C., Sinescu C. (2017). 3D Printing of a Mandibular Bone Deffect. Mater. Plast..

[13] Gabor A., Zaharia C., Todericiu V., Szuhanek C., Cojocariu A.C., Duma V.F., Sticlaru C., Negrutiu M.L., Antoniac I.V., Sinescu C. (2018). Adhesion of Scaffolds with Implants to the Mandibular Bone with a Defect. Mater. Plast..

[14] Pina S., Ribeiro V.P., Marques C.F., Maia F.R., Silva T.H., Reis R.L., Oliveira J.M. (2019). Scaffolding Strategies for Tissue Engineering and Regenerative Medicine Applications. Materials.

[15] Gao C., Deng Y., Feng P., Mao Z., Li P., Yang B., Deng J., Cao Y., Shuai C., Peng S. (2014). Current Progress in Bioactive Ceramic Scaffolds for Bone Repair and Regeneration. Int. J. Mol. Sci..

[16] Lopez-Alvarez M., Rodriguez-Valencia C., Serra J., Gonzales P. (2013). Bio-inspired ceramics: Promising scaffolds for bone tissue engineering. Procedia Eng..

[17] Sabree I., Gough J.E., Derby B. (2015). Mechanical properties of porous ceramic scaffolds: Influence of internal dimensions. Ceram. Int..

[18] Ghassemi T., Shahroodi A., Ebrahimzadeh M.H., Mousavian A., Movaffagh J., Moradi A. (2018). Current Concepts in Scaffolding for Bone Tissue Engineering. Arch. Bone Jt. Surg..

[19] Vitale-Brovarone C., Verne E., Robiglio L., Martinasso G., Canuto R.A., Muzio G. (2008). Biocompatible glass–ceramic materials for bone substitution. J. Mater. Sci. Mater. Med..

[20] Bruschi G.B., Crespi R., Capparè P., Bravi F., Bruschi E., Gherlone E. (2013). Localized Management of Sinus Floor Technique for Implant Placement in Fresh Molar Sockets. Clin. Implant. Dent. Relat. Res..

[21] Scarano A., Degidi M., Iezzi G., Pecora G., Piattelli M., Orsini G., Caputi S., Perrotti V., Mangano C., Piattelli A. (2006). Maxillary Sinus Augmentation with Different Biomaterials: A Comparative Histologic and Histomorphometric Study in Man. Implant. Dent..

[22] Ferrini F., Capparé P., Vinci R., Gherlone E.F., Sannino G. (2018). Digital versus Traditional Workflow for Posterior Maxillary Rehabilitations Supported by One Straight and One Tilted Implant: A 3-Year Prospective Comparative Study. BioMed Res. Int..

[23] Gherlone E.F., Ferrini F., Crespi R., Gastaldi G., Capparé P. (2015). Digital Impressions for Fabrication of Definitive “All-on-Four” Restorations. Implant. Dent..

[24] Cattoni F., Chirico L., Merlone A., Manacorda M., Vinci R., Gherlone E.F. (2021). Digital Smile Designed Computer-Aided Surgery versus Traditional Workflow in “All on Four” Rehabilitations: A Randomized Clinical Trial with 4-Years Follow-Up. Int. J. Environ. Res. Public Health.

[25] Szuhanek C.A., Watz C.G., Avram Ș., Moacă E.-A., Mihali C.V., Popa A., Campan A.A., Nicolov M., Dehelean C.A. (2020). Comparative Toxicological In Vitro and In Ovo Screening of Different Orthodontic Implants Currently Used in Dentistry. Materials.

[26] Chen Z., Patwari M., Liu D. (2019). Cytotoxicity of orthodontic temporary anchorage devices on human periodontal ligament fibroblasts in vitro. Clin. Exp. Dent. Res..

[27] Coelho C.C., Grenho L., Gomes P.S., Quadros P.A., Fernandes M.H. (2019). Nano-hydroxyapatite in oral care cosmetics: Characterization and cytotoxicity assessment. Sci. Rep..

[28] Chen Q.Z., Thompson I.D., Boccaccini A.R. (2006). 45S5 Bioglass^®^-derived glass-ceramic scaffolds for bone tissue engineering. Biomaterials.

[29] Alves C.B.B., Segurado M.N., Dorta M.C.L., Dias F.R., Lenza M.G., Lenza M.A. (2016). Evaluation of cytotoxicity and corrosion resistance of orthodontic mini-implants. Dent. Press J. Orthod..

[30] Mohammed I., Alwahab Z.N. (2017). An Evaluation of the Effect of Artificial Saliva with Different pH on Shear Bond Strength of Veneering Ceramic to Metal and Zirconia Substructure (In Vitro Study). WJPR.

[31] Arai T., Beckhoff B., Kanngieber B., Langhoff N., Wedell R., Wolff H. (2006). Introduction. Handbook of Practical X-ray Fluorescence Analysis.

[32] Oladebeye A.O. (2017). Assessment of Heavy Metals in Nigerian Vegetables and Soils in Owo and Edo Axes Using X-Ray Fluorescence (Xrf) Technique.

[33] West M., Ellis A.T., Potts P.J., Streli C., Vanhoof C., Wegrzynek D., Wobrauschek P. (2010). Atomic spectrometry update-X-Ray fluorescence spectrometry. J. Anal. Atomic Spectrom..

[34] Thrivikraman G., Madras G., Basu B. (2014). In vitro/In vivo assessment and mechanisms of toxicity of bioceramic materials and its wear particulates. RSC Adv..

[35] Moacă E.-A., Farcaș C., Coricovac D., Avram S., Mihali C.-V., Drăghici G.-A., Loghin F., Păcurariu C., Dehelean C. (2019). Oleic Acid Double Coated Fe_3_O_4_ Nanoparticles as Anti-Melanoma Compounds with a Complex Mechanism of Activity—In Vitro and In Ovo Assessment. J. Biomed. Nanotechnol..

[36] Moacă E.-A., Farcaș C., Ghițu A., Coricovac D., Popovici R., Cărăbă-Meiță N.-L., Ardelean F., Antal D.S., Dehelean C., Avram Ș. (2018). A Comparative Study of Melissa officinalis Leaves and Stems Ethanolic Extracts in terms of Antioxidant, Cytotoxic, and Antiproliferative Potential. Evid. Based Complement. Alternat. Med..

[37] Maghiari A.L., Coricovac D., Pinzaru I.A., Macașoi I.G., Marcovici I., Simu S., Navolan D., Dehelean C. (2020). High Concentrations of Aspartame Induce Pro-Angiogenic Effects in Ovo and Cytotoxic Effects in HT-29 Human Colorectal Carcinoma Cells. Nutrients.

[38] Interagency Coordinating Committee on the Validation of Alternative Methods (ICCVAM) (2010). ICCVAM-Recommended Test Method Protocol: Hen’s Egg Test—Chorioallantoic Membrane (HET-CAM) Test Method. ICCVAM Test. Method Eval. Rep..

[39] Popa A., Dehelean C., Calniceanu H., Watz C., Brad S., Sinescu C., Marcu O.A., Popa C.S., Avram S., Nicolov M. (2020). A Custom-Made Orthodontic Mini-Implant—Effect of Insertion Angle and Cortical Bone Thickness on Stress Distribution with a Complex In Vitro and In Vivo Biosafety Profile. Materials.

[40] Luepke N.P. (1985). Hen’s egg chorioallantoic membrane test for irritation potential. Food Chem. Toxicol..

[41] Navalón C., Ros-Tárraga P., Murciano A., Velasquez P., Mazón P., De Aza P.N. (2019). Easy manufacturing of 3D ceramic scaffolds by the foam replica technique combined with sol-gel or ceramic slurry. Ceram. Int..

[42] Theocharidou A., Tsioptsias C., Konstantinidou K., Kontonasaki E., Sivropoulou A., Panayotou C., Paraskevopoulos K.M., Koidis P. (2011). Human PDL Fibroblasts Proliferation in Scaffolds on Bioactive Glass Modified Ceramics. Bioceram. Dev. Appl..

[43] Gherlone E.F., Capparé P., Tecco S., Polizzi E., Pantaleo G., Gastaldi G., Grusovin M.G. (2016). A Prospective Longitudinal Study on Implant Prosthetic Rehabilitation in Controlled HIV-Positive Patients with 1-Year Follow-Up: The Role of CD4+ Level, Smoking Habits, and Oral Hygiene. Clin. Implant. Dent. Relat. Res..

[44] Bretcanu O., Samaille C., Boccaccini A.R. (2008). Simple methods to fabricate Bioglass^®^-derived glass–ceramic scaffolds exhibiting porosity gradient. J. Mater. Sci..

[45] Barbeck M., Unger R., Witte F., Wenisch S., Schnettler R. (2017). Xenogeneic bone grafting materials. Int. Mag. Oral Implant..

[46] Montazerian M., Zanotto E.D., Boccaccini A.R., Brauer D.S., Hupa L. (2017). Chapter 2—Bioactive Glass-ceramics: Processing, Properties and Applications. Bioactive Glasses: Fundamentals, Technology and Applications.

[47] ISO 22442-1:2020 Medical Devices Utilizing Animal Tissues and Their Derivatives—Part 1: Application of Risk Management. https://www.iso.org/standard/74280.html.

[48] ISO 22442-2:2020 Medical Devices Utilizing Animal Tissues and Their Derivatives—Part 2: Controls on Sourcing, Collection and Handling. https://www.iso.org/standard/74281.html.

[49] ISO 22442-3:2007 Reviewed and Confirmed in 2015, Medical Devices Utilizing Animal Tissues and Their Derivatives—Part 3: Validation of the Elimination and/or Inactivation of Viruses and Transmissible Spongiform Encephalopathy (TSE) Agents. https://www.iso.org/standard/39351.html.

[50] Lee H.J., Yi G.S., Lee J.W., Kim D.J. (2017). Physicochemical characterization of porcine bone-derived grafting material and comparison with bovine xenografts for dental applications. J. Periodontal. Implant. Sci..

[51] Guo J.L., Piepergerdes T.C., Mikos A.G., Alghamdi H., Jansen J. (2020). Chapter 6—Bone graft engineering: Composite scaffolds. Dental Implants and Bone Grafts.

[52] Woloszyk A., Buschmann J., Waschkies C., Stadlinger B., Mitsiadis T.A. (2016). Human Dental Pulp Stem Cells andGingival Fibroblasts Seeded into Silk Fibroin Scaffolds Have the Same Ability in Attracting Vessels. Front. Physiol..

[53] Kilic K., Kesim B., Sumer Z., Polat Z., Kesim S. (2013). In vitro cytotoxicity of all-ceramic substructural materials after aging. J. Dent. Sci..

[54] ISO 10993-5:2009 Reviewed and Confirmed in 2017, Biological Evaluation of Medical Devices—Part 5: Tests for In Vitro Cytotoxicity. ISO Catalogue, Edition 3. https://www.iso.org/standard/36406.html.

[55] Lee M., Hwang J.H., Lim K.M. (2017). Alternatives to in Vivo Draize Rabbit Eye and Skin Irritation Tests with a Focus on 3D Reconstructed Human Cornea-like Epithelium and Epidermis Models. Toxicol. Res..

[56] De Oliveira C.A., Dario M.F., Sarruf F.D., Mariz I.F.A., Velasco M.V.R., Rosado C., Baby A.R. (2016). Safety and Efficacy Evaluation of Gelatin-Based Nanoparticles Associated with UV Filters. Coll. Surf. B Biointerfaces.

[57] Öztürk A.A., Kıyan H.T. (2020). Treatment of Oxidative Stress-Induced Pain and Inflammation with Dexketoprofen Trometamol Loaded Different Molecular Weight Chitosan Nanoparticles: Formulation, Characterization and Anti-Inflammatory Activity by Using in Vivo HET-CAM Assay. Microvasc. Res..

[58] Ardlin B.I., Dahl J.E., Tibballs J.E. (2005). Static Immersion and Irritation Tests of Dental Metal-Ceramic Alloys. Eur. J. Oral Sci..

