# Supplementary material for: Scaffold-Type Structure Dental Ceramics with Different Compositions Evaluated through Physicochemical Characteristics and Biosecurity Profiles
Source: Materials (Basel). 2021 Apr 27;14(9):2266. doi: 10.3390/ma14092266 (PMC8124461; doi:10.3390/ma14092266)
Supplement: Supplementary file 1 [file materials-14-02266-s001.zip › materials-1182060-supplementary.pdf]

materials MDPI

Supplementary materials

Scaffold-Type Structure Dental Ceramics with Different Compositions Evaluated through Physicochemical Characteristics and Biosecurity Profiles

Mihai M.C. Fabricky [1,†], Alin-Gabriel Gabor [2,3,†], Raluca Adriana Milutinovici [4], Claudia Geanina Watz [5,6,*], Ștefana Avram [6,7], George Drăghici [6,8], Ciprian V. Mihali [9,10], Elena-Alina Moacă [6,8], Cristina Adriana Dehelean [3,6,8], Atena Galuscan [11,12], Roxana Buzatu [13,*], Virgil-Florin Duma [14,15], Meda-Lavinia Negrutiu [2,3] and Cosmin Sinescu [2,3]

1 Department of Prosthodontics, Faculty of Dental Medicine, Victor Babeș University of Medicine and Pharmacy, 9 Revolutiei 1989 Ave., 300070 Timisoara, Romania; fabricky@me.com

2 Department of Prostheses Technology and Dental Materials, Faculty of Dental Medicine, Victor Babes University of Medicine and Pharmacy of Timisoara, 9 Revolutiei 1989 Ave., 300070 Timisoara, Romania; alin.gabor@umft.ro (A.-G.G.); negrutiu.meda@umft.ro (M.-L.N.); minosinescu@gmail.com (C.S.)

3 Research Center in Dental Medicine Using Conventional and Alternative Technologies, Timisoara, 9 Revolutiei 1989 Ave., 300070 Timisoara, Romania; cadehelean@umft.ro (C.A.D.)

4 Department of Orthodontics, Faculty of Dental Medicine, Victor Babeș University of Medicine and Pharmacy, 9 Revolutiei 1989 Ave., 300070 Timisoara, Romania; raluca_balan22@yahoo.com

5 Department of Pharmaceutical Physics, Faculty of Pharmacy, Victor Babeș University of Medicine and Pharmacy, 2nd Eftimie Murgu Sq., 300041 Timișoara, Romania

6 Research Center for Pharmaco-Toxicological Evaluations, Faculty of Pharmacy, "Victor Babes" University of Medicine and Pharmacy, Eftimie Murgu Square No. 2, 300041 Timisoara, Romania; stefana.avram@umft.ro (Ș.A.); draghici.george-andrei@umft.ro (G.D.); alina.moaca@umft.ro (E.-A.M.)

7 Department of Pharmacognosy, Faculty of Pharmacy, Victor Babeș University of Medicine and Pharmacy, 2nd Eftimie Murgu Sq., 300041 Timișoara, Romania

8 Departament of Toxicology, Faculty of Pharmacy, Victor Babeș University of Medicine and Pharmacy, 2nd Eftimie Murgu Sq., 300041 Timișoara, Romania;

9 Department of Life Sciences, Faculty of Medicine, Vasile Goldis Western University of Arad, 86 No., Liviu Rebreanu St., 310414 Arad, Romania; mihaliciprian@yahoo.com

10 Molecular Research Department, Research and Development Station for Bovine, 32 No., Bodrogului St., 310059 Arad, Romania

11 Department of Preventive Dentistry, Faculty of Dental Medicine, Victor Babeș University of Medicine and Pharmacy, 14A Tudor Vladimirescu Ave., 300173 Timisoara, Romania; atedent@yahoo.com

12 Translational and Experimental Clinical Research Center in Oral Health (TEXC-OH), 14A Tudor Vladimirescu Ave., 300173 Timisoara, Romania

13 Department of Dental Aesthetics, Faculty of Dental Medicine, Victor Babeș University of Medicine and Pharmacy, 300041 Timișoara, Romania

14 3OM Optomechatronics Group, Faculty of Engineering, "Aurel Vlaicu" University of Arad, 77 Revolutiei Ave., 310130 Arad, Romania; dumavirgil@yahoo.co.uk

15 Faculty of Mechanical Engineering, Polytechnic University of Timisoara, 1 Mihai Viteazu Ave., 300006 Timisoara, Romania

* Correspondence: farcas.claudia@umft.ro (C.G.W.); drbuzaturoxana@gmail.com (R.B.)

† contributed equally to this work.

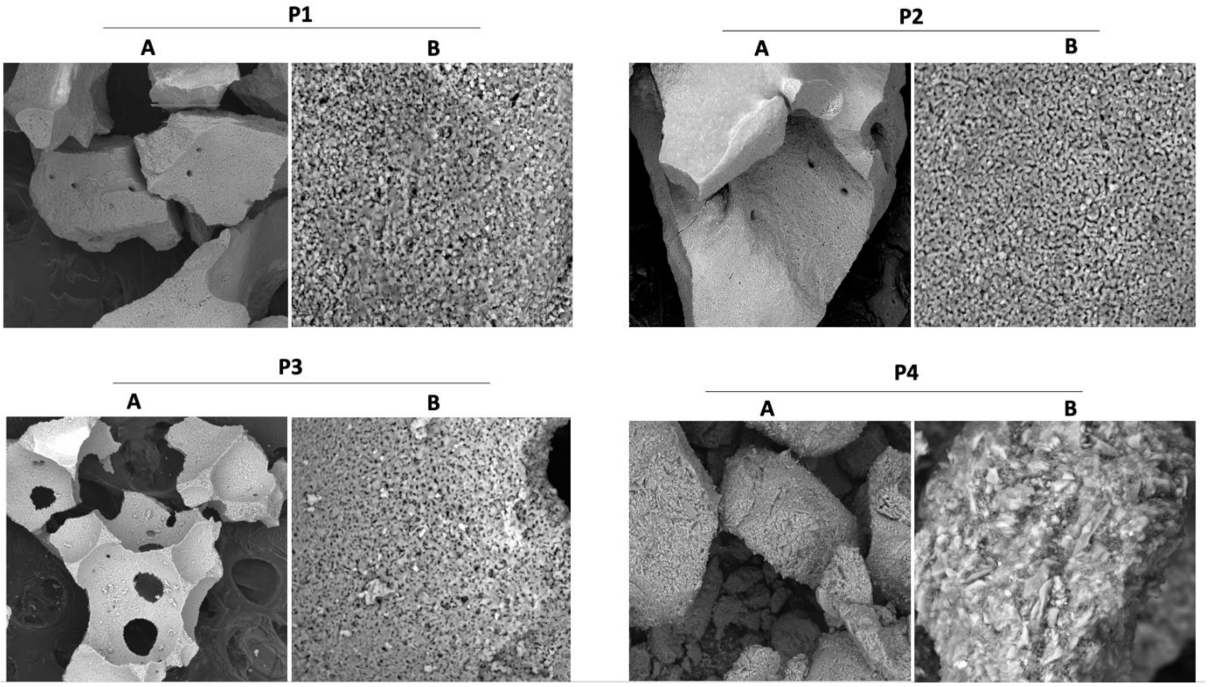

Figure S1. SEM micrographs of the ceramic samples (P1, P2, P3, P4) after 8 days immersion in acidic saliva (pH = 3.393) at 37 ^{0}C and 260 RPM with on-off shaking protocol; **A** – represents the general aspects of the samples and **B** – represents the ultrastructure details of the samples.

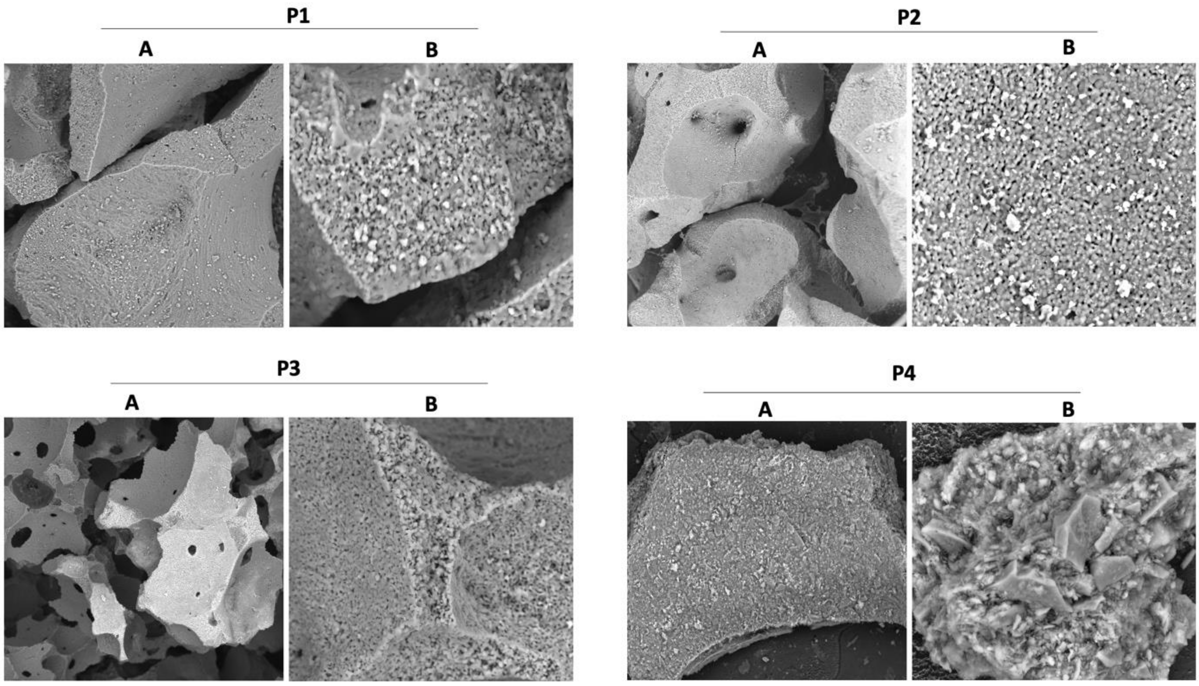

Figure S2. SEM micrographs of the ceramic samples (P1, P2, P3, P4) after 8 days immersion in neutral saliva (pH = 7.355) at 37 °C and 260 RPM with on-off shaking protocol; **A**–represents the general aspects of the samples and **B**–represents the ultrastructure details of the samples.

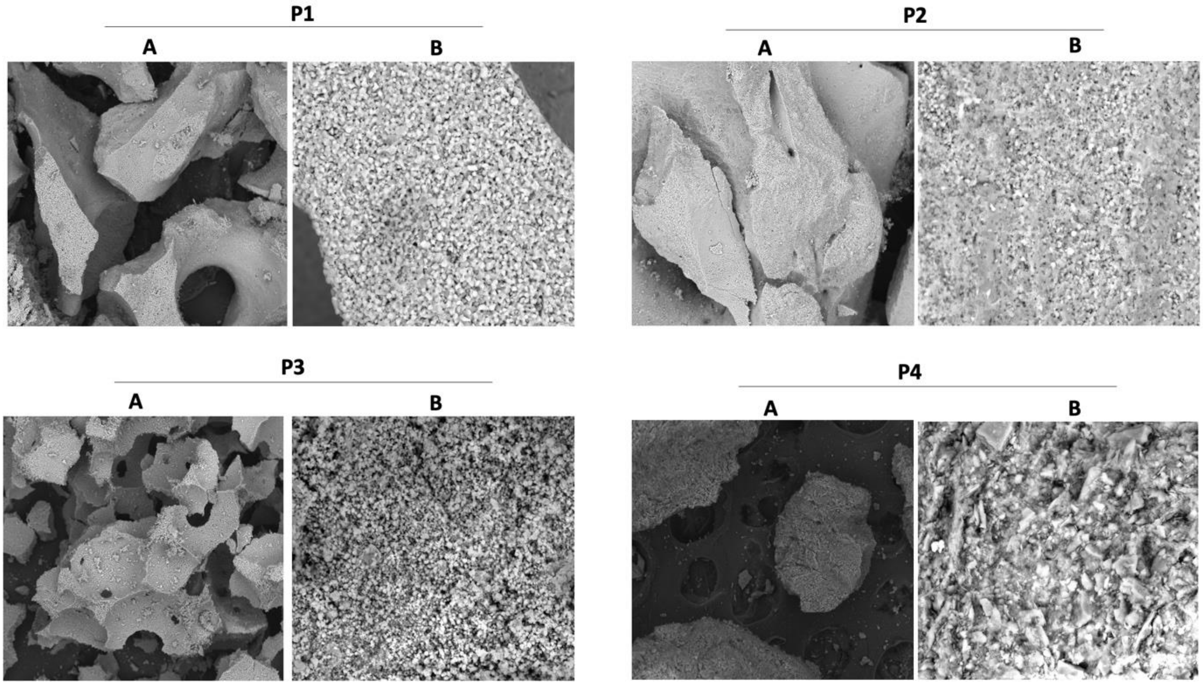

Figure S3. SEM micrographs of the ceramic samples (P1, P2, P3, P4) after 8 days immersion in alkaline saliva (pH = 10.769) at 37 °C and 260 RPM with on-off shaking protocol; **A**–represents the general aspects of the samples and **B**–represents the ultrastructure details of the samples.
